# Teaching transference focused psychotherapy to South African mental health practitioners

**DOI:** 10.4102/sajpsychiatry.v30i0.2315

**Published:** 2024-11-15

**Authors:** Henk S. Temmingh, Iliana Fanidi, Craig Bracken, Tennyson Lee

**Affiliations:** 1 Department of Psychiatry and Mental Health, Faculty of Health Sciences, University of Cape Town, Cape Town, South Africa; 2 Department of Psychiatry, University Hospital of Heraklion PAGNI, Crete, Greece; 3 Department of Psychiatry, Faculty of Health Science, University of the Witwatersrand, Johannesburg, South Africa; 4 DeanCross Personality Disorder Service, East London NHS Foundation Trust, London, United Kingdom; 5 Centre for the Understanding of Personality Disorder (CUSP), London, United Kingdom

**Keywords:** personality disorders, training, transference focused psychotherapy, attitudes, confidence

## Abstract

**Background:**

Personality disorders (PDs) are estimated to occur in 6.8% of South Africans and in 45% to 80% of clinical populations. Mental health practitioners often harbour negative attitudes and lack confidence in working with such patients. Brief training in transference focused psychotherapy (TFP) has been shown to improve attitudes and confidence in the management of clinical encounters with PD.

**Aim:**

This study aimed to describe the characteristics of attendees at a brief TFP training workshop and determine the impact of training on attitudes and clinical confidence towards patients with PD.

**Setting:**

We conducted two 3 h online workshops, spaced 1 week apart to staff at South African University training hospitals.

**Methods:**

At baseline, participants (*N* = 41) completed questionnaires on demographics, perceived need for training, supervision adequacy and perceived confidence. At baseline and after the second session, the Attitude to Personality Disorder Questionnaire (APDQ) and the Clinical Confidence in Personality Disorder Questionnaire (CCPDQ) were completed. Longitudinal data were analysed using linear mixed-effects regression.

**Results:**

In the completer sample (*N* = 13), there were significant improvements in the APDQ enthusiasm subscale (*p* = 0.029) and in clinical confidence (CCPDQ) (*p* = 0.032). The APDQ total and other subscales also showed improvements. Participants with higher baseline confidence were more likely to drop out.

**Conclusion:**

Brief training in TFP can lead to significant improvements in attitude and confidence in managing patients with PD.

**Contribution:**

This is the first study in the South African context demonstrating the potential value of brief teaching in TFP.

## Introduction

Personality disorders (PDs) occur frequently in the general population with prevalence estimates ranging from 9% to 12%.^1,2^ In turn, among psychiatric treatment seeking populations, PDs occur in as many as 45% of outpatients and even in higher numbers among inpatients.^3,4^ The most recent estimate, dating back to 2008, from the South African Stress and Health Study (SASH) determined that PDs may occur in as many as 6.8% of the South African general population, with cluster B PDs (borderline, narcissistic, antisocial and histrionic) showing a higher odds of seeking treatment from psychiatrists.^5^ Despite this high prevalence, mental health professionals often feel ill equipped to manage clinical encounters with these patients.^6^ Clinical interactions with these patients are often described as invoking difficult feelings for clinicians, such as feeling frustrated, inadequate, challenged, uncertain on how to respond, leading to views of borderline personality disorder (BPD) patients being manipulative, chaotic and creating conflict in treatment teams.^7^ More concerning even is the finding that negative attitudes may in fact increase as trainees in psychiatry progress to more advanced stages of training, the latter possibly being related to group mentality and exposure to negative attitudes, particularly around suicidal patients with PDs.^8^ Some but not all studies demonstrate that psychiatric nurses, psychiatrists and psychologists may differ in their level of negative attitudes and empathy towards these patients and other factors such as degree of exposure to this patient population, level of experience and training may also impact attitudes towards this group.^6,9,10^ A lack of confidence in utilising therapeutic techniques as well as negative attitudes may impact and negatively affect patient care for this often stigmatised and highly vulnerable group of patients.^11^ One important factor that has been demonstrated to improve attitudes is training and education in psychotherapy and PDs.^6^ In this respect, even brief courses have improved confidence and attitudes towards PD patients.^12,13,14^

Transference focused psychotherapy (TFP) is an evidence-based treatment with demonstrated efficacy in patients with PDs.^15,16,17^ Transference focused psychotherapy is derived from psychoanalytical theory and technique but modified for patients with borderline and other PDs. It is a high intensity treatment, typically delivered twice weekly (or once weekly in certain public health settings, i.e., United Kingdom National Health Service [UK NHS]) over 12–24 months.^18^ Central to this treatment is the modified tactical use of the therapy frame that includes goal setting, tight boundaries about attendance and acting-out behaviour, the management of secondary gains and possible re-contracting. These tactics all contribute to the playing out of the negative transference within the therapeutic relationship, which becomes a focus of treatment. Working with a neutral stance and from surface to depth, TFP focusses on using techniques such as clarification of mental states and experiences of self and perception of others with the aim of increasing mentalisation (an ability to interpret the intentions and behaviours of both self and others in terms of intentional mental states, feelings, thoughts, beliefs and desires). As therapy progresses, clarification may be followed by empathic confrontation of discrepant self and other states. Therapist-centred interpretations (where the focus is on the patient’s view of what is going on in the therapist’s mind and how her words and behaviour may be experienced by the patient, rather than any attempt to link this to being a projection of the patient’s)^19^ are used in the earlier stages of therapy, progressing to patient-centred interpretations (addressing what the patient is doing, wishing or thinking) as the therapy advances. These interpretations are centred around the dominant in-session affect and related transference patterns and object relational dyads (internalisation of important attachment figures shaping views of the self and others). Ultimately, these techniques are utilised with the strategic aim to foster greater integration of split-off dissociated ways of relating to self and others.^20,21^ The application of TFP principles in the management of transference and countertransference, the latter which is often evoked with BPD patients even during regular clinical consultations in outpatient clinics or emergency rooms, has been demonstrated to provide a useful approach to such clinical encounters.^22^ Training in applied TFP for psychiatric registrars in the UK has also been shown to improve attitudes and understanding of PD as well as confidence in managing countertransference and transference during clinical encounters.^23^

We set out to provide a brief training workshop in basic TFP principles in managing difficult clinical encounters in routine clinical settings to mental health practitioners (students and supervisors) at training hospitals and clinics attached to university training centres across South Africa. Firstly, we aimed to describe the characteristics, attitudes and confidence in the management of PDs of psychiatry registrars and supervisors attending transference focused psychotherapy training, as well as the factors associated with attrition from training. Secondly, we aimed to determine if there is an improvement in attitudes and clinical confidence in the management of PDs from before to after receiving training in TFP. We expected to find an increase in attitudes and clinical confidence from before and after receiving training.

## Research methods and design

### Population and sample

We conducted a training evaluation in a group of mental health practitioners attending a brief training workshop in TFP. In May 2023, a series of two 3-h online training sessions, spaced 1 week apart, were held in the theory and clinical application of TFP in the management of BPD. The training was held as part of the South African College of Psychiatrists (Colleges of Medicine of South Africa) online training initiative. Attendees at the training were mental health practitioners working at training sites and were eligible to participate in the study if they were affiliated with a university as a trainee or supervisor. University affiliated trainees and staff typically include registrars in psychiatry, consultant psychiatrists, clinical psychologists and intern clinical psychologists. In South Africa, specialisation in psychiatry is minimum 4-year qualification (post medical internship) that includes 1 year of theoretical teaching in psychology and psychotherapy (forming one paper out of three as the Part-I, FCPsych exam), followed by training on PD as part of the Part-II FCPsych course. Mandatory clinical training in psychotherapy includes two ‘short cases’ (6–8 session cases in Cognitive Behavioural Therapy (CBT) or Dialectical Behaviour Therapy [DBT] group-skills training), and one ‘long case’ (minimum 20 sessions in psychodynamic or supportive psychotherapy), with regular (minimum of 30 h) supervision from a psychologist or psychiatrist. In turn, clinical psychology is a 6-year masters training, that includes a final year clinical internship.

### Procedures and measures

Each workshop was started by an introduction to the basic theory of TFP, including object-relations theory, working in the transference and countertransference, concepts of technical neutrality and techniques of clarification, confrontation and interpretation. Each theory session was followed by a live role-play of a fictional patient with borderline personality presenting with suicidality in the context of an emergency room and then at a community clinic. The workshop was structured around a freely accessible educational paper. (Lee, T. and Hersh, R.G. 2019).^24^

Eligible participants who gave informed consent completed a baseline questionnaire prior to the start of the first training session. Demographic and background characteristics (clinical training background, university affiliation, level of experience, past training) were collected. At baseline we also collected data using a 6-point Likert scale on three measures, namely the belief in need for specific training in management of BPD (BNST), experiences of the perceived adequacy of supervision and support in working with BPD (EPASS), as well as perceived confidence in the diagnosis and management of BPD (PCDM). At baseline prior to the first training session and again 1 week later after the second training session, participants completed the Attitude to Personality Disorder Questionnaire (APDQ), a validated 35-item scale that measures positive and negative attitudes towards PD.^25^ The APDQ consist of a total score and 5 subscales, measuring one positively phrased attitude (enjoyment vs. loathing), and four reverse-coded, negatively phrased attitudes (security vs. vulnerability, acceptance vs. rejection, purpose vs. futility, and enthusiasm vs. exhaustion). In addition, together with the APDQ (pre-training and 1 week later after training), participants completed the Clinical Confidence in Personality Disorder Questionnaire (CCPDQ).^26^ This questionnaire measures various aspects around confidence in the theory and skills using a psychodynamic framework for the management of PD, particularly as relevant to TFP. This scale includes items measuring confidence in conveying a diagnosis of BPD and narcissistic personality disorder (NPD), treatment contracts, objectives and frame, psychological mechanisms in PD, working with countertransference and transference, object-relations theory of PD, use of interpretations and technical neutrality. The CCPDQ is a Likert-type questionnaire and scored on 13-items with ‘never’, ‘seldom’, ‘occasionally’, ‘often’, ‘very often’, ‘always’, with higher average scores indicating greater confidence. The CCPDQ scale has demonstrated face validity,^23^ but formal validation research is still in progress.

### Statistical analysis

We inspected data for normality using frequency histograms and the Shapiro–Wilk’s test for normality. For determining internal consistency and reliability, we used the Cronbach’s alpha-coefficient. For correlations between continuous variable, we used the Pearson’s correlation coefficient for normal data and the Spearman’s rank correlation coefficient for non-normal data. We explored if there were any predictors of attrition from the study using univariate and multivariate logistic regression analysis (variables reaching significance at *p* < 0.05 in univariate models were entered into a multivariable model). For longitudinal, panel data (pre-training session to post-training session APDQ and CCPDQ scales), we used random effects linear regression with adjustment for baseline scores and maximum likelihood estimation. The final models were visually inspected for normality of residuals and outliers. We used Stata version 16 and reported results as statistically significant with a *p*-value of *p* < 0.05.^27^

### Ethical considerations

All participants provided written informed consent. Ethical clearance to conduct this study was obtained from the University of Cape Town Human Research Ethics Committee (No. HREC REF: 787/2022). Data were anonymised upon collection.

## Results

### Sample characteristics

The first training session (baseline) was attended by *n* = 110 participants and the second session, 1 week later by *n* = 64 participants. The baseline questionaries were completed by *n* = 41 participants and the follow-up session by *n* = 22 participants. Data were matched for *n* = 13 pairs who completed both baseline and follow-up questionnaires. Table 1 contains the demographic and background characteristic of the participants. Just under half of the participants were psychiatric registrars, followed by consultant psychiatrists and clinical psychologists. Just over a third of participants supervised other clinicians in the management of BPD. A quarter of participants (24.3%, *n* = 10) had attended other courses on the management of PDs. Among the 10 participants who had attended prior courses, 40% (*n* = 4) attended DBT training, 20% (*n* = 2) Schema Therapy training, 20% (*n* = 2) Other Psychodynamic (Masterson Disorders of the Self Course), 10% (*n* = 1) TFP, 10% (*n* = 1) General Psychiatric Management (GPM) and 10% (*n* = 1) ‘Other’ PD courses.

**TABLE 1:** Characteristics of participants.

Variable	Baseline (*n* = 41)		Completer (*n* = 13)	
	*N*	%	*N*	%
**Age**				
≤ 35 years	18	43.9	6	46.2
36–45 years	13	31.7	3	23.1
> 45 years	10	24.4	4	30.7
**Gender**				
Male	11	26.8	3	23.1
Female	30	73.2	10	76.9
**Practitioner type**				
Psychiatry registrar	19	46.3	7	53.9
Clinical psychologist	13	31.7	4	30.8
Consultant psychiatrist	7	17.1	2	15.3
Psychology intern	2	4.2	-	-
**Clinical supervisor (BPD)** ^a^				
Yes	15	36.6	2	15.4
No	26	63.4	11	84.6
**Level of clinical experience**				
< 4 years	15	36.6	5	38.4
4–10 years	13	31.7	4	30.8
> 10 years	13	31.7	4	30.8
**Affiliation**				
University of the Witwatersrand	17	41.5	3	23.1
University of Cape Town	8	19.5	4	30.8
University of the Free State	8	19.5	4	30.8
University of Pretoria	3	7.3	-	-
University of KwaZulu-Natal	2	4.9	-	-
University of Limpopo	2	4.9	1	7.7
Unspecified	1	2.4	1	7.7
**Attendance of prior training courses**				
Yes	10	24.4	3	23.1
No	31	75.6	10	76.9

BPD, borderline personality disorder.

a , Supervisors of staff doing any clinical work with patients with BPD.

### Internal consistency and scale reliability

The APDQ had a high degree of internal consistency (Cronbach’s alpha = 0.96) and the CCPDQ also reached a high level of reliability (Cronbach’s alpha = 0.92).

### Factors associated with attrition between baseline and second training sessions

Attrition for study participation was high between the first (*n* = 41) and second training session (*n* = 22) and only *n* = 13 baseline questionnaires were matched after the second training session (68.3% attrition). Participants who had higher scores on their perceived adequacy of supervision and support in working with BPD, had a significantly higher odds of not completing the second training session questionnaire (unadjusted odds ratio [OR] = 2.01, *p* = 0.030, 95% confidence interval [CI] = 1.07–3.80), similarly participants who scored higher on their baseline clinical confidence in the management of BPD (CCPDQ) had a significantly higher odds of not completing the post-training assessment (unadjusted OR = 1.08, *p* = 0.047, 95% CI = 1.00–1.16). There was a trend towards significance for higher odds of drop-out for participants who were actively involved in supervising clinicians in the management of BPD (unadjusted OR = 4.77, *p* = 0.068, 95% CI = 0.88–25.56). No other variables were associated with drop-out. When the two-variables reaching significance at *p* < 0.05 were entered into a multivariable logistic model, only perceived adequacy of supervision reached a trend towards significance (adjusted OR = 1.90, *p* = 0.057, 95% CI = 0.97–3.68).

### Changes in attitudes and confidence in management of borderline personality disorder

Table 2 contains the full baseline (*n* = 41) and paired data (*n* = 13) for participants completing pre- and post-study assessments. At baseline, there was a moderately strong positive and statistically significant correlation between the APDQ total score and the CCPDQ score (*r* = 0.47, *p* = 0.002). For matched participants (*n* = 13), the mean baseline total score on the APDQ was 18.38 (95% CI = 16.22–20.52), which increased to 18.97 (95% CI = 16.97–20.98), a non-significant increase baseline adjusted analysis (β = 0.59, *p* = 0.173, 95% CI = -0.26–1.45). Similarly, there were no significant improvements in the APDQ subscales measuring Enjoyment, Security, Acceptance or Purpose. There was a statistically significant improvement on the APDQ Enthusiasm subscale, with a mean increase from 3.07 to 3.42 (β = 0.35, *p* = 0.029, 95% CI = 0.03–0.65). For confidence in management of PDs using a psychodynamic approach as measured by the CCPDQ, there was a statistically significant improvement in the baseline adjusted analysis, with an increase in mean scores from 2.7 pre-training to 3.0 post-training (β = 0.31, *p* = 0.032, 95% CI = 0.03–0.59).

**TABLE 2:** Baseline and paired data for attitudes and confidence in management of borderline personality disorder.

Variable	Baseline (*n* = 41)				Completer (*n* = 13)					
	Pre-training				Pre-training				Post-training	
	Mean	± s.d.	Median	± IQR	Mean	± s.d.	Median	± IQR	Mean	± s.d.
**APDQ**										
Total score	18.5	3.5	-	-	18.4	3.5	-	-	18.9	3.3
Enjoyment	3.1	0.9	-	-	3.0	0.9	-	-	3.2	0.9
Security	4.2	0.7	-	-	4.2	0.6	-	-	4.1	0.6
Acceptance	4.2	0.9	-	-	4.2	1.0	-	-	4.3	0.7
Purpose	4.0	0.9	-	-	3.9	0.9	-	-	3.9	0.8
Enthusiasm	2.9	0.8	-	-	3.0	0.7	-	-	3.4*	0.7
CCPDQ total	3.1	0.8	-	-	2.7	0.7	-	-	3.0*	0.6
EPASS	3.8	1.2	-	-	3.2	1.3	-	-	-	-
PCDM	4.2	0.8	-	-	3.9	0.9	-	-	-	-
BNST	-	-	6	1	-	-	6	1	-	-

APDQ, Attitude to Personality Disorder Questionnaire; CCPDQ, Confidence in Personality Disorder Questionnaire; EPASS, Experiences of the Perceived Adequacy of Supervision and Support in working with BPD; PCDM, Perceived Confidence in the Diagnosis and Management of BPD; BNST, Belief in Need for Specific Training in management of BPD; s.d., standard deviation; IQR, interquartile range.

* , *p* < 0.05.

## Discussion

In this study, we have shown that relatively brief training in TFP is associated with a small but significant improvement in attitudes and confidence in the management of PDs. In the completer analysis, both scales measuring attitude and confidence tended to be unchanged or had small, improved scores. We did not find any significant improvement in the total score for attitude as measured by the APDQ total score, or the enjoyment, security, acceptance, purpose subscales. However, we found a small but significant increase in the enthusiasm subscale of the APDQ. Improvement in enthusiasm is important as this sub-score measures levels of frustration and feelings of being drained (i.e., exhaustion) and brief training was able to reduce such negative emotions around potential encounters with personality disordered patients. Also, we found a small significant improvement in the clinical confidence in managing PD using TFP principles.

Our findings are in line with those found in the TFP training workshop study conducted with UK trainee psychiatrists, where an improvement in the clinical confidence using TFP principles as measured by the CCPDQ improved significantly.^23^ However, in contrast to our findings in the study conducted by Sinisi et al., the APDQ total/composite score also increased.^23^ Similarly in a recent Italian study (unpublished, personal communication), the authors also found an increase in the APDQ total score, as well as in the APDQ enthusiasm subscale, the latter being a finding very similar to ours. In addition, these findings are similar to that found in training psychiatry registrars in mentalisation-based treatment or MBT (a related psychodynamic treatment for PDs) where the authors found an increase in the enthusiasm subscale for the APDQ, but in contrast to our finding for the ‘purpose’ and total APDQ subscales.^28^ There may be several reasons for these differences. Firstly, our study population included both trainees (registrars in psychiatry and psychology interns) as well as their supervisors (psychiatrists and clinical psychologists), as opposed to the two London and Italian studies which were aimed at psychiatry registrars. In contrast to the Italian study and London MBT-teaching studies for which teaching took place in person, our training was entirely delivered in an online mode.

## Conclusion

This study has important limitations. Study dropout was high and the small sample size in the completer analysis may have impacted the results, making it potentially difficult to detect smaller effects. Our drop-out rate is consistent with similar training workshop studies in GPM for PD, using multiple time-points, where attrition has been as high as 73%.^13^ In analysing factors associated with dropout in adjusted analyses, there was a trend for participants who perceived that they have received adequate supervision for this patient group to be more likely to drop out. Also, in unadjusted analysis supervisors were more likely to drop out at a trend level of significance, while those who had a high level of confidence at baseline were also significantly more likely to drop out. This might suggest that supervisors and more senior clinicians were more likely to drop-out. Other factors for drop-out may be related to external factors. Our workshop was held country-wide across different training sites and affiliated universities, and did not necessarily coincide with the routine registrar training-day timeslots, and thus clinical obligations may have precluded attendance at two separate sessions. Another important limitation is the use of the Clinical Confidence in Personality Disorder scale, which has not been studied in a formal validation study. Of note, we measured internal consistency and found high Cronbach’s alpha coefficients for the CCPDQ in this sample. As our study was conducted online, this mode of delivery could have affected participation during training and influenced our results. We cannot comment on the longer-term impact of our training, as our assessment was conducted immediately after training. Furthermore, as our population included a heterogenous group of practitioners with different levels of clinical experience, we cannot generalise our findings to other trainings using more homogenous populations. Lastly, we did not include a control group, therefore we are not able to comment on how specific our findings are to TFP training.

Our findings are encouraging and suggest that future training of clinicians in TFP principles may be useful in developing a better understanding of PDs, equipping practitioners with more skills and confidence to manage difficult clinical encounters. However, to evaluate this, future training may need to be delivered in person, targeted at more homogenous populations, and be administered over a single extended length session across multiple settings, or ensure conditions conducive to higher attendance if delivered over multiple sessions.
